# Online Prediction of Health Care Utilization in the Next Six Months Based on Electronic Health Record Information: A Cohort and Validation Study

**DOI:** 10.2196/jmir.4976

**Published:** 2015-09-22

**Authors:** Zhongkai Hu, Shiying Hao, Bo Jin, Andrew Young Shin, Chunqing Zhu, Min Huang, Yue Wang, Le Zheng, Dorothy Dai, Devore S Culver, Shaun T Alfreds, Todd Rogow, Frank Stearns, Karl G Sylvester, Eric Widen, Xuefeng Ling

**Affiliations:** ^1^ College of Computer Science and Technology Zhejiang University Hangzhou China; ^2^ HBI Solutions, Inc Palo Alto, CA United States; ^3^ Stanford University Stanford, CA United States; ^4^ Shanghai Children's Hospital Shanghai Jiao Tong University Shanghai China; ^5^ HealthInfoNet Portland, ME United States

**Keywords:** health care costs, electronic medical record, prospective studies, statistical data analysis, risk assessment

## Abstract

**Background:**

The increasing rate of health care expenditures in the United States has placed a significant burden on the nation’s economy. Predicting future health care utilization of patients can provide useful information to better understand and manage overall health care deliveries and clinical resource allocation.

**Objective:**

This study developed an electronic medical record (EMR)-based online risk model predictive of resource utilization for patients in Maine in the next 6 months across all payers, all diseases, and all demographic groups.

**Methods:**

In the HealthInfoNet, Maine’s health information exchange (HIE), a retrospective cohort of 1,273,114 patients was constructed with the preceding 12-month EMR. Each patient’s next 6-month (between January 1, 2013 and June 30, 2013) health care resource utilization was retrospectively scored ranging from 0 to 100 and a decision tree–based predictive model was developed. Our model was later integrated in the Maine HIE population exploration system to allow a prospective validation analysis of 1,358,153 patients by forecasting their next 6-month risk of resource utilization between July 1, 2013 and December 31, 2013.

**Results:**

Prospectively predicted risks, on either an individual level or a population (per 1000 patients) level, were consistent with the next 6-month resource utilization distributions and the clinical patterns at the population level. Results demonstrated the strong correlation between its care resource utilization and our risk scores, supporting the effectiveness of our model. With the online population risk monitoring enterprise dashboards, the effectiveness of the predictive algorithm has been validated by clinicians and caregivers in the State of Maine.

**Conclusions:**

The model and associated online applications were designed for tracking the evolving nature of total population risk, in a longitudinal manner, for health care resource utilization. It will enable more effective care management strategies driving improved patient outcomes.

## Introduction

Health care spending in the United States has grown rapidly since the 1980s [1]. The total health care expenditures for 2012 were more than US $2.8 trillion, accounting for more than 17% of the gross domestic product [2]. It is estimated that this figure will reach US $3.1 trillion in 2014 [3] and become the largest component of the federal budget by 2015 [4]. The trend of increasing spending on health care demands focused attention, which should include analyzing health care resource utilization drivers and predicting future care resource utilization. An effective prediction of future resource utilization can help improve care resource allocation and care delivery, supporting the transition from a volume-based incentive system to a value-based system. To accomplish this transition, health care organizations [5] will need to meet the multiple medical needs of patients while achieving improved outcomes at reduced expense [6].

A variety of factors can affect resource utilization, such as patient age, care providers, medical technologies, and morbidity of patients [7]. Several statistics-based algorithms and methodologies have been developed to forecast future health care expenditures [8-19]. However, many of these studies had limitations caused by incomplete data resources or research targeted only on a particular subgroup of patients, such as age- [14,19] or disease-specific [9,19] populations. Similarly, many of the existing commercial models for resource utilization prediction were constructed using insurance claim data methodologies [20]. Lack of validation of prospective data was another weakness in some studies [19].

The goal of our study was to develop a population-based predictive model to estimate the risk of health care resource utilization in next 6 months for patients in Maine using the state’s health information exchange (HIE) electronic medical record (EMR)-based data system. The ability of a health care provider organization to effectively predict health care resource utilization risks using only EMR data is important in the shifting US health care payment system. Provider systems continue to extend their EMR infrastructure throughout their acute, subacute, and physician provider network capturing greater longitudinal clinical patient histories in their EMR. Further, provider systems are entering into more value- and risk-based contracts creating a need for more predictive and proactive care strategies. Using EMR data solely for predictive model development has 2 benefits: (1) it negates the need for integrating claims data from multiple payer sources which is costly and (2) the EMR data are in real time providing more timely information than latent claims data systems and risk models that are typically 60 to 90 days old by the time a provider receives the information. The data for our study were provided by the HIE in Maine, which contains clinical histories and demographic information derived from EMRs for more than 1 million patients covering all payers, all diseases, and all age groups in Maine. The predictive risk model was constructed by statistically learning the correlations between the 6-month total health care utilization and the preceding 12-month demographic and clinical data. It was validated prospectively on both an individual level and a population (per 1000 patients) level. Applications of the model in analyzing and managing health care resource utilization were explored.

We hypothesized that past 12-month EMR-based clinical histories of patients can be used to predict risks of their next 6-month resource utilization via statistical learning from all Maine HIE patient data contained in the statewide HIE of longitudinal patterns. To empower the visualization and exploration of the total population risks of more than 1 million patients in the State of Maine, online applications were architected, aiming to connect in real time, aggregate and centrally integrate data, and to compute the next 6-month risks for population health management. To our knowledge, this study is the first to predict future health care resource utilization using only EMR data at the patient level across an entire state.

## Methods

### Ethics Statements

This work was done under a business arrangement between HealthInfoNet (HIN) and HBI Solutions, Inc (HBI). Use of the data is governed by the business associate agreement between HIN and HBI. No protected health information was released for the purpose of research. Instead, HBI implemented their application, which was the foundation of the agreement, and then reported on the findings resulting from applying this model to the products that HIN now deploys in the field.

Because this study analyzed deidentified data, the Stanford University Institutional Review Board considered it exempt (October 16, 2014).

### Data Warehouse

We constructed a data warehouse consisting of all the Maine HIE’s aggregated patient histories. Data elements included patient demographic information, laboratory tests and results, radiographic procedures, medication prescriptions, and diagnoses and procedures, which were coded according to the *International Classification of Diseases, 9th Revision, Clinical Modification* (*ICD-9-CM*). Diagnoses were further clustered into 190 chronic diagnoses according to the Clinical Classifications Software (CCS) developed at the Agency for Healthcare Research and Quality (AHRQ). The CCS-coded diagnosis was used to determine the presence of chronic conditions for each patient. Census data from the US Department of Commerce Census Bureau were integrated into our data warehouse to provide an approximation of patients’ socioeconomic status information in terms of the average household mean/median family income and average level of educational attainment distinguished by the zip code of each patient. Missing data handling is described in Multimedia Appendix 1. Our model initially contained 14,680 elements (features) describing the patient’s full clinical histories. The feature dimension was significantly reduced in the subsequent feature selection process for modeling purposes.

### Cohort Construction

The study covered patients visiting any HIN-connected facility from January 1, 2009, to December 31, 2013. To qualify for the study, all patients included were alive and resided in Maine.

A retrospective cohort of 1,273,114 patients, represented by clinical information between January 1, 2012, and December 31, 2012, was assembled to develop a model to predict the risk of health care resource utilization between January 1, 2013, and June 30, 2013. This model was validated by a prospective cohort of 1,358,153 patients with clinical information between July 1, 2012, and June 30, 2013, used to predict the health care resource utilization risk from July 1, 2013, to December 31, 2013. Cohort construction details are shown in Multimedia Appendix 2. Patient demographics of the 2 cohorts are shown in Table 1.

**Table 1:** Cohort characteristics.

Characteristic		Cohort	
		Retrospective (01/01/12-12/31/12) n=1,273,114	Prospective (07/01/12-06/30/13) n=1,358,153
**Gender, n (%)**			
	Female	669,021 (52.55)	710,042 (52.28)
	Male	604,093 (47.45)	648,111 (47.72)
Age (years), median (IQR)		43.71 (22.40-60.87)	43.76 (22.83-61.11)
Family income estimate (US $), median (IQR)		59,209 (49,148-68,589)	58,984 (49,148-68,082)
**Education, median (IQR)**			
	Percent high-school graduate or higher	90.50 (87.30-93.20)	90.40 (87.20-92.80)
	Percent bachelor’s degree or higher	24.40 (17.90-33.00)	23.90 (17.90-31.30)

### Next Six-Month Health Care Resource Utilization Scoring Metric Development

The resource utilization depends on much more than just cost. Mean outpatient, emergency department (ED), and inpatient days are more accurate reflection of the trending of health care resource utilization. The national mean cost for different types of resource utilization was used as the weighting mechanism in our computational analysis. Patient’s health care utilization was calculated using mean costs associated with specific encounter types (outpatient, ED, and inpatient days) derived from a national database of historical encounter- and inpatient day-based costs [21,22]. This method created an overall single utilization measure per patient across the varying encounter types. This utilization measure was used as the outcome to predict.

We targeted to predict future risks of patient health care resource utilizations. Future resource utilization distribution analysis on the retrospective cohort revealed a nonlinear correlation between the future resource utilization and the corresponding population sizes. As shown in Multimedia Appendix 3, a small proportion of patients consumed a relatively large amount of health care resources (1.79%, 22,850/1,273,114, of the total population took up 45.96%, US $509.97 million/US $1109.53 million of the next 6-month resource utilization), whereas a large proportion of patients consumed insignificant health care services (62.56%, 796,503/1,273,114, of the population accounted for 2.86%, US $31.74 million/US $1109.53 million of the next 6-month resource utilization). The highly concentrated health care resource utilization distribution of our data indicates that the future utilization characteristics were largely varied among patients of different health statuses, thus revealing the necessity to stratify patients based on the predicted future resource utilization level and treat them separately.

Retrospectively, each patient’s next 6-month health care resource utilization was assigned a score ranging from 0 to 100 that correlated with the percentile of the next 6-month resource utilization for that patient in the retrospective cohort. Therefore, a decision tree-based predictive model could be trained with selected preceding 12-month EMR clinical information as independent variables and the next 6-month utilization scores as the dependent variable. The derived predictive algorithm forecasts future risk scores indicative of next 6-month health care resource utilization for each patient.

### Model Development

**Figure 1:**
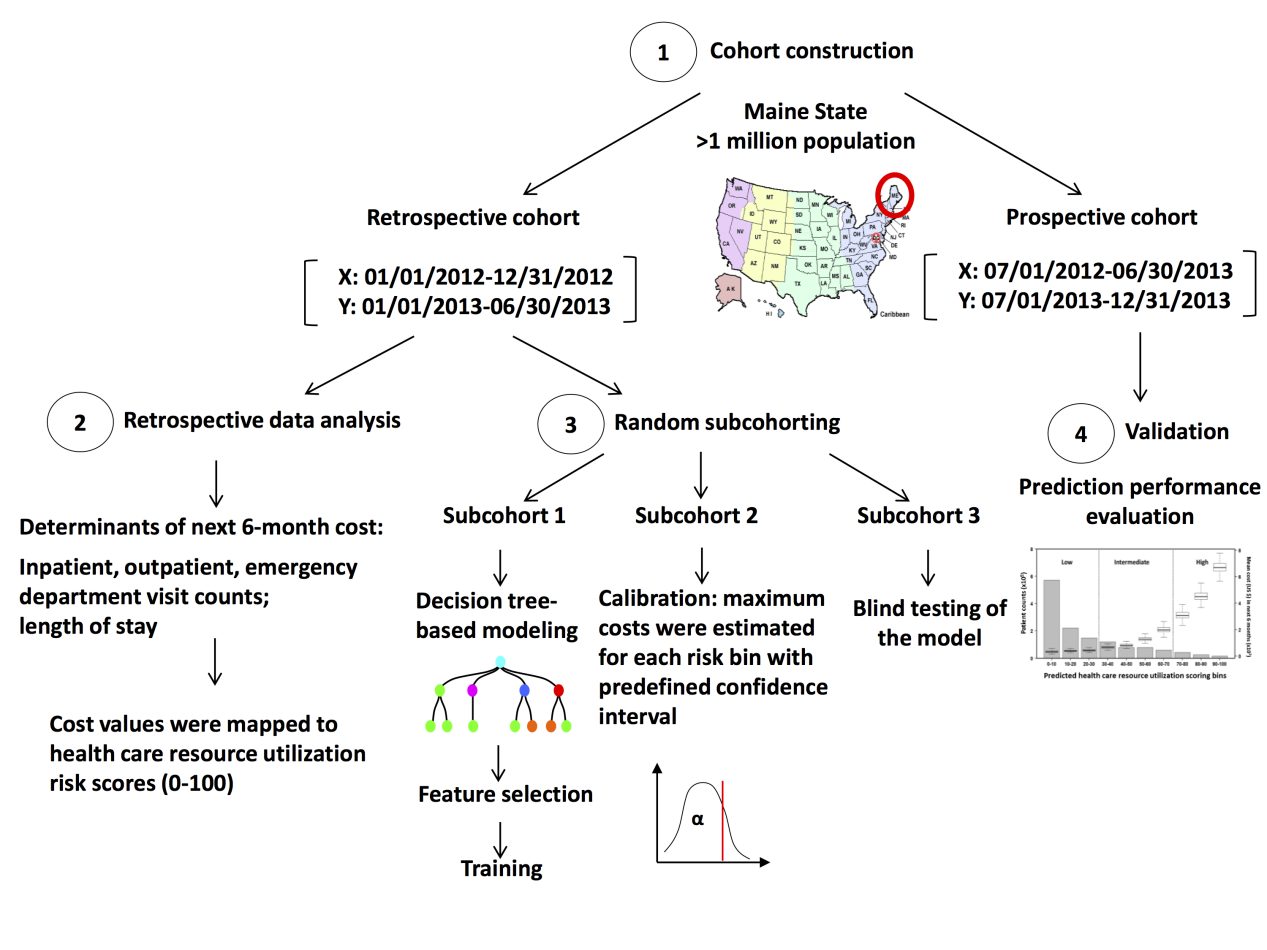
Study design to develop the next 6-month health care resource utilization predictive algorithm. Maine HIE data were split into retrospective and prospective cohorts based on different time frames. A decision tree–based model estimated the health care resource utilization risks in the next 6 months by statistically learning the preceding 12-month clinical histories and were trained, calibrated, and blind tested with the retrospective cohort. The predictive risk model was then validated with the prospective cohort.

Patients in the retrospective cohort were randomly partitioned into 3 subcohorts (Figure 1) for model training, calibration, and blind testing purposes. The model was then validated with the prospective cohort.

#### Training

Random forest methodology [23-25] was applied to construct 300 decision trees to generate predicted risks based on the preceding 12-month clinical history. Specifically, each of the 300 trees was grown using a randomly selected 63.20% (268,203/424,371) of the patients and one third of the clinical features in the training subcohort. At each node, trees were split by choosing a split feature value producing the minimum sum of square, which equaled the sum of square difference between the risk score of each patient and the mean risk score of all patients on the daughter node to which the patient was assigned. Trees were grown until the size of each terminal node was less than 5. Final decisions were calculated by averaging the predicted risks of each tree.

#### Calibration

The predictive algorithm derived from the training subcohort was applied on the calibrating subcohort to obtain the risks of patients in that subcohort. The risks were then grouped into 10 bins (0-10, 10-20,..., 90-100), with each bin mapped to a unique health care resource utilization. The value was set as the estimated maximum next 6-month resource utilization of patients falling into a specific bin with a confidence level of alpha. The value of alpha was defined as the proportion of patients in that bin whose next 6-month resource utilization was less than the associated maximum resource utilization value.

The total population was then stratified into 3 main levels indicating an estimation of the level of health care resource utilization for each patient in the next 6 months: high (risk ≥70), intermediate (30≤ risk <70), and low (risk <30). The thresholds (30 and 70) were chosen arbitrarily. Therefore, our analysis produced 2 risk measures: a continuous risk score ranging from 0 to 100 and a categorical risk defined by 3 levels. The former was applied for numerical performance tests whereas the latter was used for stratified analysis in the model validation process, including health care resource utilization analysis and clinical pattern analysis in the model validation process.

#### Feature Selection

To reduce the calculation complexity of the modeling process, we applied a feature selection process. A total of 2000 features with top variations were first selected from 14,680 initial features to train a random forest model. A list of the features and importance was generated from the random forest model. Second round modeling was done thereafter by using the top 10, 20, 30, 40, 50, 60, 70, 80, 90, and 100 features from the feature list. A best ensemble model was chosen according to the performance of sensitivity and confidence level across all risk levels. Our statistical learning finally identified 70 variables predictive of next 6-month resource utilization risk. These variables were segmented by demographic groups (n=7), different encounter history (n=40), care facilities (n=8), primary and secondary diagnoses (n=14), and outpatient prescription medications (n=1; Table 2).

**Table 2:** Electronic medical record features used to develop the model.

Feature group	Feature description (12-month clinical history from January 1, 2012-December 31, 2012)
Encounter history (n=40)	Visit counts of different encounter types (emergency/outpatient/inpatient/preadmission)
	The accumulated length of hospitalized stay
	Historical resource utilization
	Counts of historical chronic disease diagnoses
	Counts of total and no redundant total laboratory tests and outpatient prescriptions
Demographics (n=7)	Income, education, payer
	Age group was defined by age on January 1, 2013 (0, 1-5, 6-12, 13-18, 19-34, 35-49, 50-65, and ≥65 years)
Facility (n=8)	Different facilities
Diagnosis (n=14)	Counts for primary diagnosis and secondary diagnosis
Outpatient prescriptions (n=1)	Counts for different outpatient prescriptions

#### Blind Testing

After calibration, the model’s performance was blind tested with the blind testing subcohort. Again, we applied the calibrated model to each patient in that subcohort to derive the predictive risks, grouped all the patients into the 10 bins defined in the calibration process, and identified the proportion of the patients whose resource utilization was less than the estimated maximum resource utilization in each bin.

### Model Validation

The predictive algorithm was tested on an independent prospective cohort to validate its effectiveness of risk stratification. The model performance was evaluated on both individual patient level and population level by measuring the confidence levels of prediction and mean resource utilization per person at different risk levels. Clinical patterns of patients at each risk level were analyzed to explore potential applications of the model. A case study was performed to measure the consistency between our risk prediction and resource utilization in a given period (see Multimedia Appendix 4).

### Using the Model to Analyze Chronic Disease Care Resource Utilization

The health care resource utilization associated with the presence of chronic disease diagnosed in the preceding 12 months was analyzed using the proposed model. Chronic conditions were defined using the AHRQ Chronic Condition Indicator [26], which provides an effective way to categorize *ICD-9-CM* diagnosis codes into 1 of 2 categories: chronic and nonchronic. The mean resource utilization and population sizes were summarized for all the chronic diseases identified by the CCS coding system. LOESS regression was applied to analyze the correlations between the resource utilization and risk stratification for each chronic disease category.

### Online Population Explorer: Statewide Real-Time Surveillance of Population Risks

The risk model and associated online real-time application were designed to track the evolving nature of total population risk of resource utilization in a longitudinal manner. The predictive algorithm was applied to the individual’s discriminating feature data extracted from a patient-level database to risk stratify the patients. Individual data were then aggregated for population exploration of resource utilization risks. Results were visualized on an online dashboard. The technical details of our online population explorer implementation are described in Multimedia Appendix 5.

## Results

### Prospective Performance: Confidence Levels, Mean Resource Utilization, and Risk Stratification

Prospective performance was gauged by the confidence level of prediction and the mean resource utilization per patient in 10 distinctive bins (Table 3). The confidence levels remained at a fairly high level, fluctuating between 0.723 and 0.889 for all risk levels. It illustrated that the predicted risks associated with the estimated maximum resource utilization had an acceptable accuracy for individual patients regardless of their risk levels (ie, resource utilization).

**Table 3:** Prospective results of our risk model predictive of next 6-month resource utilization (from July 1, 2013, to December 31, 2013).

Result statistics	Predicted risk bin									
	Low			Intermediate				High		
	0-10	10-20	20-30	30-40	40-50	50-60	60-70	70-80	80-90	90-100
Patients, n (%)^a^	571,538 (42.08)	220,746 (16.25)	147,853 (10.89)	119,242 (8.78)	79,152 (5.83)	78,585 (5.79)	59,134 (4.35)	41,711 (3.1)	25,264 (1.86)	14,928 (1.10)
Estimated maximum resource utilization (US $)	0	170	340	510	680	925	1870	2720	4625	13,301
Resource utilization per person^b^ (US $), mean (SD)	353.69 (5539.44)	425.86 (7034.20)	449.89 (3194.92)	690.62 (7982.95)	868.47 (11,386.03)	1315.39 (6624.14)	2087.44 (14,347.49)	3211.58 (20,286.97)	4530.99 (12,796.93)	6823.42 (21,814.22)
Confidence level^c^	0.784	0.735	0.805	0.783	0.754	0.723	0.805	0.790	0.796	0.889

^a^ Patient percentage of each risk bin is defined as the percentage of patients in that bin of the total prospective population.

^b^ Mean resource utilization per person in each risk bin is defined as the next 6-month mean resource utilization per person in that bin.

^c^ Confidence level of each risk bin is defined as the proportion of patients in that bin with next 6-month resource utilization less than the estimated maximum resource utilization.

A monotonic increase was found in mean next 6-month resource utilization per patient from low- to high-risk levels from July 1, 2013, to December 31, 2013 (Table 3). In summary, at low-, intermediate-, and high-risk levels, each patient costs a mean US $385.76, US $1124.33, and US $4276.88 in the next 6 months, respectively. *P* values of resource utilization distributions between these 3 levels were all less than .001 (one-sided Mann-Whitney test). Such findings demonstrate that our predictive model is capable of forecasting the patients who will account for either small or large proportions of next 6-month health care resource utilization.

**Figure 2:**
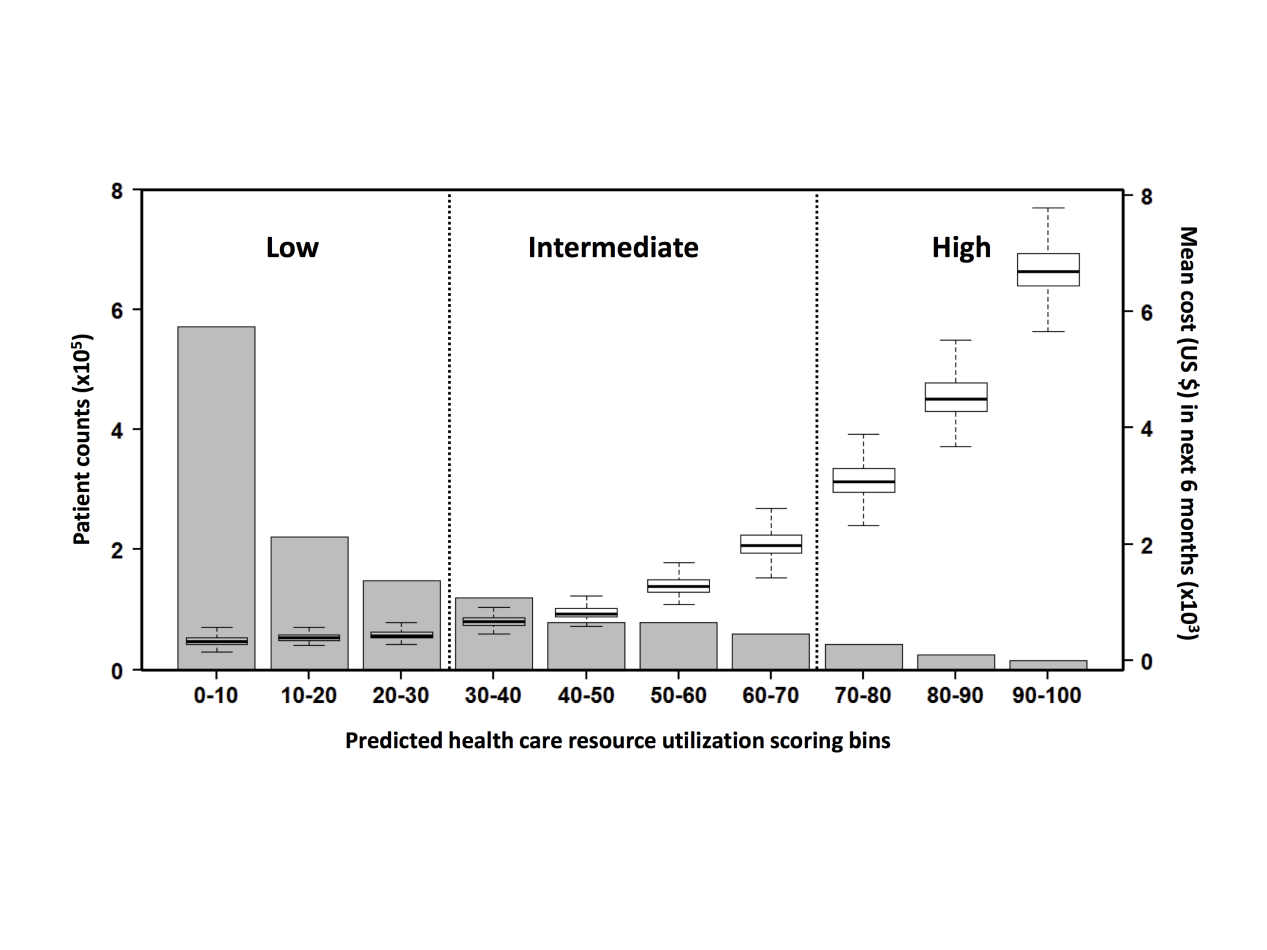
The prospective performance of the model. Prospective validation of the model: the mean next 6-month resource utilization distribution (box-and-whisker plot) and the patient counts (gray bar) versus the predicted risks. The resource utilization distributions were calculated per 1000 patients per 6 months.

The distribution of prospective next 6-month resource utilization per 1000 patients in each bin was also analyzed (Figure 2). A monotonic increase in total future spending accompanied by a monotonic decrease in patient counts as the resource utilization risk increases is displayed in the figure. As in the retrospective results, this revealed that a small proportion of patients accounted for a large proportion of the resource utilization: 571,538 patients cost a mean US $353.69, whereas 14,928 patients cost a mean US $6823.42. The box-and-whisker plot also showed that the trend of the total next 6-month resource utilization per 1000 patients correlated with the risk stratification generated by our algorithm: the predicted high resource-utilization population cost more than the predicted low resource-utilization population. It indicates that the division of the population based on the risks produced by the predictive model was intuitive, which supports the use of our model for practical applications focused on identifying patients and populations with high resource utilization for appropriate interventions.

### Clinical Patterns Associated With Risk Levels

Clinical patterns in the next 6 months of patients in the prospective cohort were summarized based on the health care resource utilization risk levels (see Multimedia Appendix 6). Despite decreases in population and total resource utilization percentages, a monotonic increase of each of the clinical patterns was found from low to high resource-utilization level, including mean resource utilization, percentage of elderly individuals (age ≥ 65 years), percentage of patients making inpatient or ED visits, and percentage of patients with chronic diseases. Patients with high risks of resource utilization were mostly elderly (41.35%, 33,865/81,903), had inpatient (28.55%, 23,381/81,903) or ED visits (45.12%, 36,958/81,903), or had chronic diseases (83.92%, 68,737/81,903). This corresponds with previous research that people who were elderly [27] and had chronic conditions [28] accounted for a large percentage of expenses. Moreover, a fair percentage of high-risk patients had hypertension (30.35%, 24,860/81,903), diabetes (21.69%, 17,764/81,903), heart disease (25.91%, 21,224/81,903), or asthma or chronic obstructive pulmonary disease (14.20%, 11,634/81,903), all of which were reported as major expensive chronic conditions [29]. In all, clinical patterns of high-risk patients identified by our EMR-based algorithm were similar to those revealed by claims data, indicating that reasonable prediction of health care resource utilization can be achieved via EMR and demographics without using any billing information.

### Resource Utilization Analysis for Patients with Chronic Diseases

**Figure 3:**
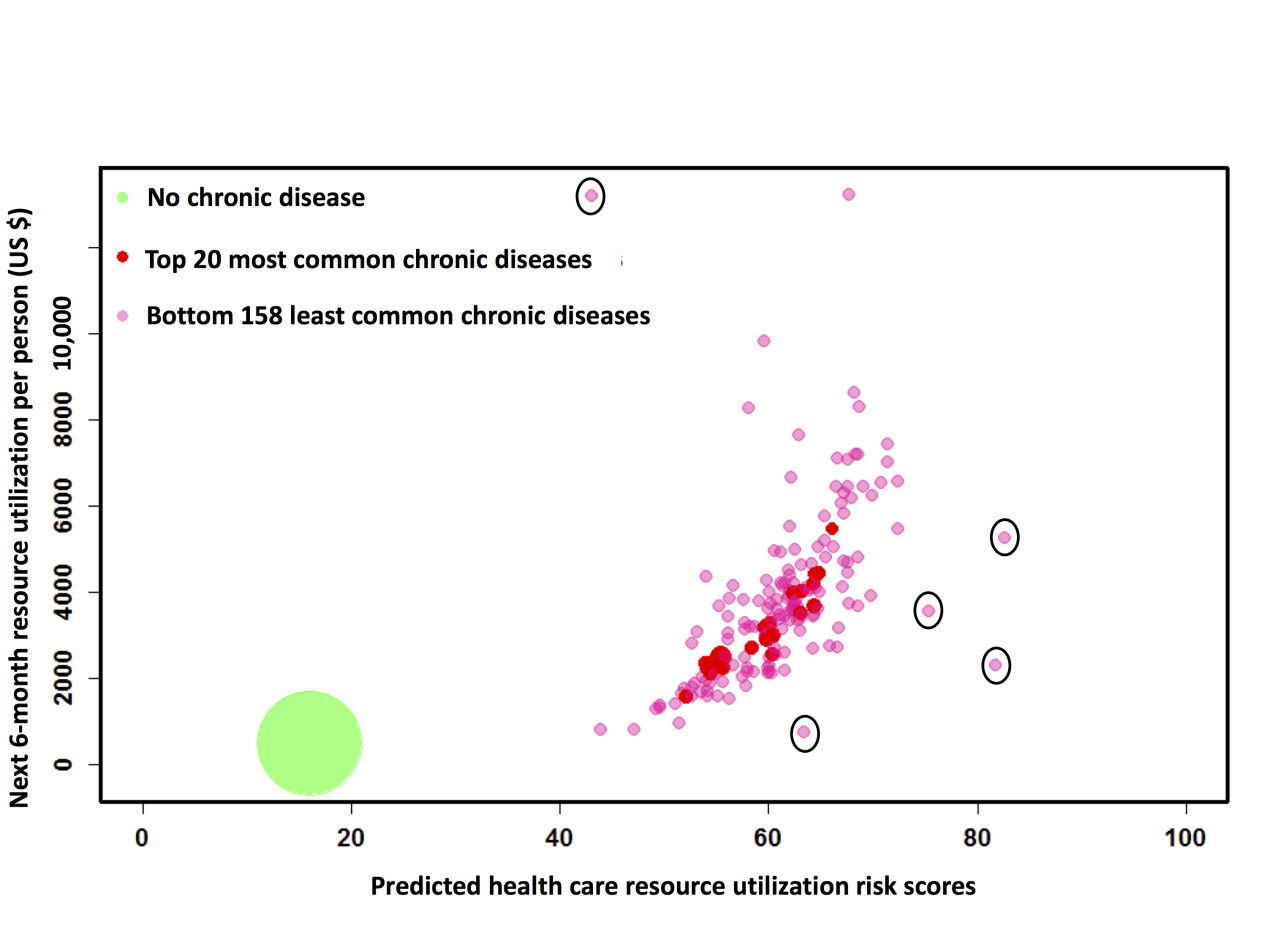
Prospective analysis of next 6-month resource utilizations stratified by chronic diseases. Bubble chart of all 178 chronic diseases (red for diseases with top 20 patient counts and pink for others) stored in our database together with the nonchronic disease group (green). Each bubble represents a chronic disease group, demonstrating mean values of the next 6-month resource utilization and the risks of the patients diagnosed with that disease. The bubble diameter is proportional to the patient counts. Outliers are marked with black circles.

The next 6-month health care resource-utilization patterns were analyzed for patients with chronic diseases (identified by the CCS coding system) in the prospective cohort. There were 178 chronic diseases in total, illustrated as bubbles in Figure 3, in which each bubble represents a patient group sharing the same chronic disease. Patients without any diagnosed chronic disease were grouped separately (the green bubble in Figure 3). The population size for each chronic disease was proportional to the bubble diameter. Figure 3 demonstrates that the algorithm effectively separated the nonchronic from the chronic disease populations. The patients without any chronic disease diagnosis had both the lowest risk (16.1) and lowest next 6-month mean resource utilization (US $490.72). The mean future resource utilization of the chronic disease groups with higher risks tended to be higher than those with lower risks. It should be noted that the outliers in Figure 3 (marked with black circles) represented diseases that only 2-11 patients had in the prospective cohort and thus can be ignored.

**Figure 4:**
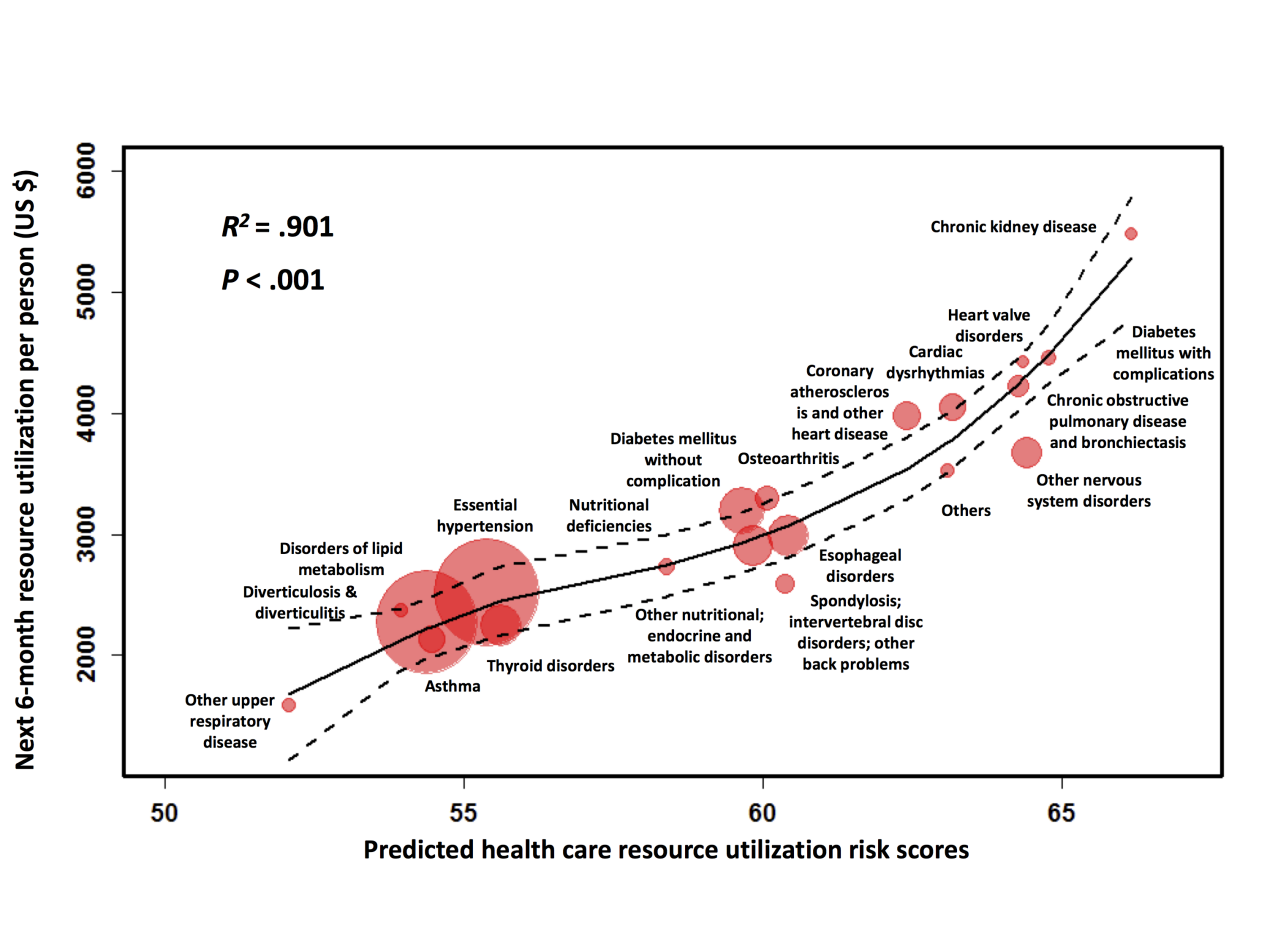
Close examination of the prospective analysis of next 6-month resource utilizations stratified by the top 20 most common chronic diseases. The relationship between the resource utilization and risk score were smoothed by LOESS regression (solid line: the fitting curve; dashed line: the 0.9 confidence level boundaries) showing a good linearity with R-squared=.901 and P <.001.

The resource utilization and predicted health care resource utilization risks for the 20 most common disease groups are shown in Figure 4. The LOESS smoothing curve, with 0.95 confidence interval boundary lines, was plotted to estimate the correlation between the mean resource utilization and the corresponding risks for each disease group. The high *R* ^*2*^ value (*R* ^*2*^=.901) and low *P* value (<.001, calculated by the Gaussian fitting method) indicated that a high linearity was achieved between the next 6-month resource utilization and the mean risk values across the top 20 chronic disease groups. Such linearity validated the effectiveness of our predictive algorithm in risk stratification on the prospective cohort. Furthermore, this semilinear correlation enabled a rough approximation of the future resource utilization for each disease by incorporating the chronic disease diagnosis information into the risk stratification.

Figure 4 illustrates that disorders of lipid metabolism and essential hypertension were 2 chronic diseases with the largest population in our database (n=110,369 and n=113,387, respectively) with mean next 6-month resource utilization of US $2273.22 and US $2512.30 per patient. Conversely, chronic kidney disease, diabetes mellitus with complications, and heart valve disorders were 3 diseases representing the highest mean next 6-month resource utilization (US $5478.18, US $4454.35, and US $4423.01 per patient, respectively), but had relatively small population sizes (n=12,835, n=15,588, and n=13,923, respectively). This confirms that a large amount of the health care resource utilization was consumed by a relatively small percentage of the overall population, most with chronic diseases.

### Online Explorer of Statewide Population Risks of Resource Utilization

**Figure 5:**
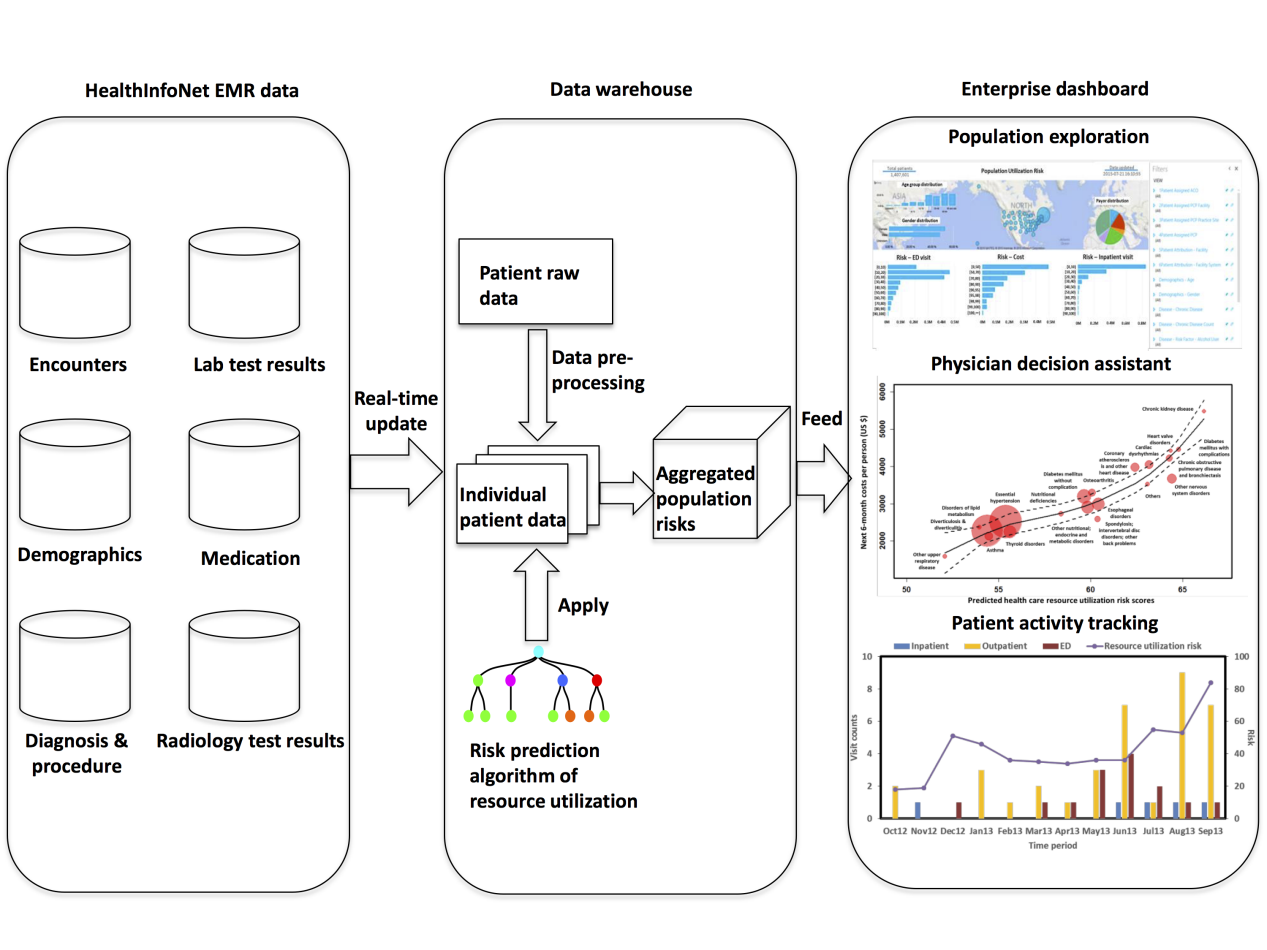
Schematic demonstration of data flow and communications of a population risk exploration system, which allows online real-time assessment of population resource utilization risk.

Our predictive analytics was integrated into the Maine State HIE system (Figure 5) to allow real-time surveillance of population risks of resource utilization. This online population risk surveillance dashboard (see Multimedia Appendix 7) empowers the Accountable Care Organization field staff and population health managers to visualize the risks derived from each resident’s historical medical records in the State of Maine. With our prospectively validated algorithm, our coherent view of population risks of resource utilization can thus be feasible to resolve the barrier of the fragmented nature of population health information to improve public health practice.

## Discussion

Extremely high expenditure rates in health care have become a nationwide issue in the United States. To approach this issue, we developed a model to predict the next 6-month health care resource-utilization risk of patients based on their past 12-month clinical histories. Prospective validation results demonstrated the effectiveness of our model in risk stratification of the future health care spending for patients with sufficient accuracy at all risk levels.

Of significant note in our research is that the proposed risk model was derived using the EMR data from the Maine HIE, the same data source as our previous studies on emergency visit and revisit risks [30,31]. The success of these models demonstrates that, with high dimensional structured data aggregation and statistical learning, past longitudinal clinical patterns can be used to forecast future resource utilization. These studies together establish an approach for developing effective clinical forecasting systems. The approach can be applied to many more use cases including predicting diseases, utilization (inpatient, pharmacy, imaging), and mortality. Integration of HIE data with predictive analytic tools enables cross-analysis and can help construct a comprehensive risk profile of the Maine population.

Our risk model was derived using EMR and demographic data only. Prospective performance (see Multimedia Appendix 6) shows that the high resource-utilization population identified by our EMR-based model had clinical patterns similar to those summarized by claims. Therefore, hospitals can reliably predict future patient resource utilization using their internal EMR and do not necessarily need to incur the resource utilization of integrating insurance claims data to achieve this. Moreover, EMR-based information is generated in near real time, whereas claims-based risk models are typically several months old by the time a provider receives the information [32]. Real-time risk scores facilitate more timely patient care.

The purpose of categorizing risk predictions was to identify high-risk patients (ie, those with relatively high probability of resource utilization). The high-risk group (risk ≥ 70) was more interesting than the other 2 groups because evidence suggests that well-organized interventions targeting high-risk patients can result in a decreased rate of admission or readmission and, therefore, significant resource savings. Further analysis on clinical patterns of high-risk patients could be used for a more personalized or precisely targeted approach to reduce future resource utilization. Furthermore, the online application tool of our model provides a tracking of patients’ activities and risks, allowing users to identify patients with increasing risks. Those patients are also of interest to clinicians and early interventions may prevent them from becoming heavy users of health care resources.

Our algorithm took special focus on the impact of chronic disease history on future resource utilization. As shown in Figure 4, chronic kidney disease, diabetes mellitus with complications, and heart valve disorders were 3 common high-impact diseases having the highest next 6-month resource utilizations and the correlated predicted risks. Such results can be explained by the fact that heart problems, diabetes, and kidney disease are commonly associated with one another and these diseases together with cancer and obesity were reported to comprise the majority of national medical spending every year [33]. Patient populations, grouped by their chronic disease diagnoses, exhibited good linearity between their future resource utilizations and their predicted risks indicating that our model is able to provide each type of chronic disease with a reasonable assessment of future health care expense. In other words, our model can give not only patient-oriented forecasts, but also disease-oriented forecasts of future resource utilization. This feature provides a direct link from our predictive algorithm to health care resource utilization because patients with long-term illness tend to account for a high volume of revisits to either inpatient or ED care settings. Knowing in advance the projected care service usage associated with chronic medical conditions can help providers make more informed decisions on the allocation of care management services with the objective of decreasing unnecessary utilization associated with treating chronically ill patients.

Although HIE data represent an ideal source of communitywide/regional patient data, operational HIEs are not present in all states. Samples collected from HIE might have unexpected bias and not match exactly the nationwide population characteristics. After overcoming these limitations, our predictive model will be improved with a broader applicability in health care globally.

Increasing health care expenditures in the United States have placed significant burdens on the national economy, calling for more cost-effective care strategies. Our study derived an EMR-based prospectively validated model to predict the health care resource utilization in the next 6 months for each of the more than 1 million patients in Maine. This model can assist care providers in applying appropriate care management services aimed at optimizing the resource allocation of caring for high-risk patients. Future studies will focus on integrating payer claims data with the HIE data to get a more accurate and timely prediction of projected future resource utilization. Having this information will assist in budgeting and resource planning in addition to supporting care management programs. The addition of claims data may also improve feature performance in the predictive model as additional or supplemental encounters, medication, diagnosis, and procedure information could be derived from claims data to fill patient longitudinal data gaps in the HIE data. The goal is to utilize the results of this study and future studies to support health care planning, financial management, and public health functions in addition to care management.

## Appendix

Multimedia Appendix 1 Missing data handling.

Multimedia Appendix 2 Retrospective and prospective cohort construction and inclusion/exclusion criteria.

Multimedia Appendix 3 Relationship between the total next 6-month resource utilization and population size in different bins in the retrospective cohort. Samples were grouped into ten bins based on their next 6-month resource utilization. In each bin, the population size (black bar) and the resource utilization (white bar) of all the samples were plotted in the form of percentages they took up in the total population or total resource utilization.

Multimedia Appendix 4 Case study of resource utilization patterns.

Multimedia Appendix 5 System implementation of the online population-based risk model for health care resource utilization.

Multimedia Appendix 6 Next 6-month clinical patterns of patients at different risk levels in the prospective cohort.

Multimedia Appendix 7 Online total population resource-utilization risk monitoring dashboard.

## Abbreviations

AHRQ: Agency for Healthcare Research and Quality
CCS: Clinical Classifications Software
ED: emergency department
EMR: electronic medical record
HIE: health information exchange
HIN: HealthInfoNet
ICD-9-CM: International Classification of Diseases, 9th Revision, Clinical Modification
