# Supplementary figures and images for: Online Prediction of Health Care Utilization in the Next Six Months Based on Electronic Health Record Information: A Cohort and Validation Study
Source: J Med Internet Res. 2015 Sep 22;17(9):e219. doi: 10.2196/jmir.4976 (PMC4642374; doi:10.2196/jmir.4976)

### Retrospective cohort

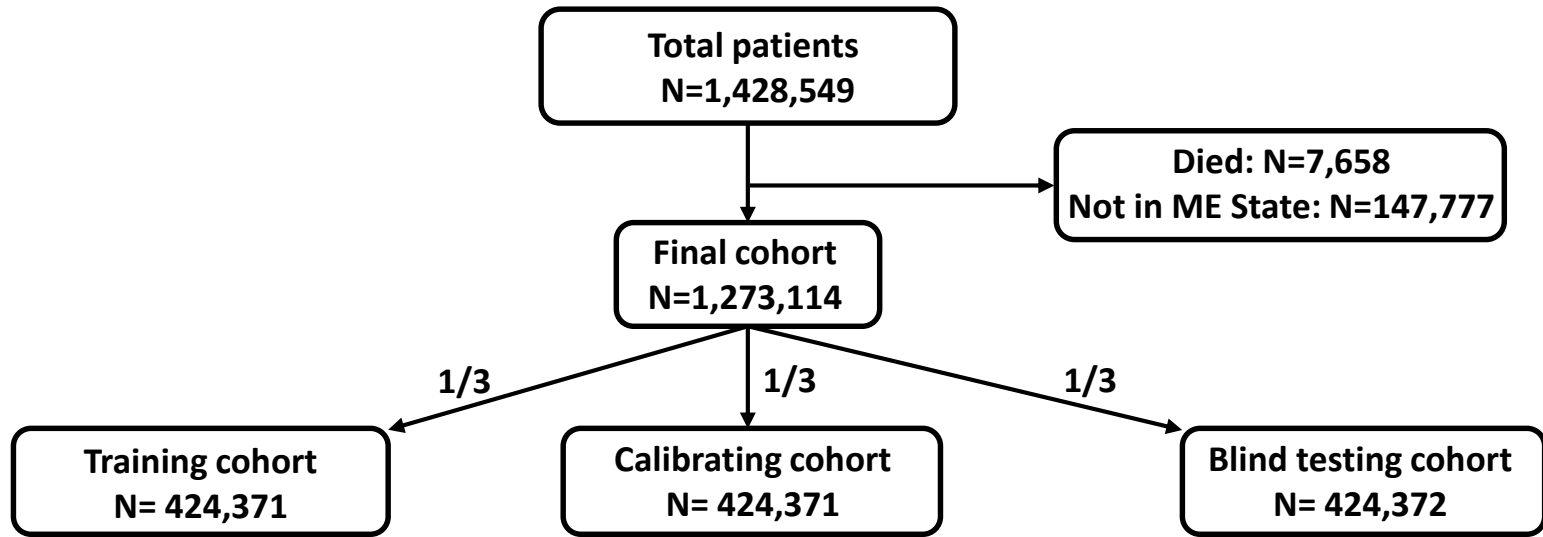

### Prospective cohort

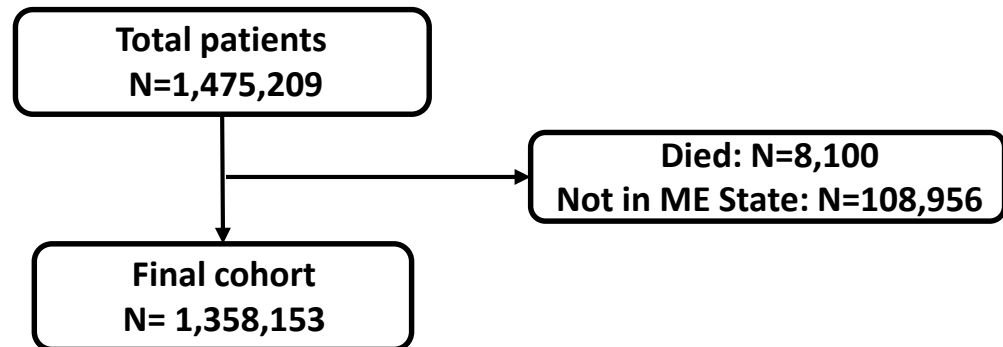

Supplement: Multimedia Appendix 2 [file jmir_v17i9e219_app2.pdf]

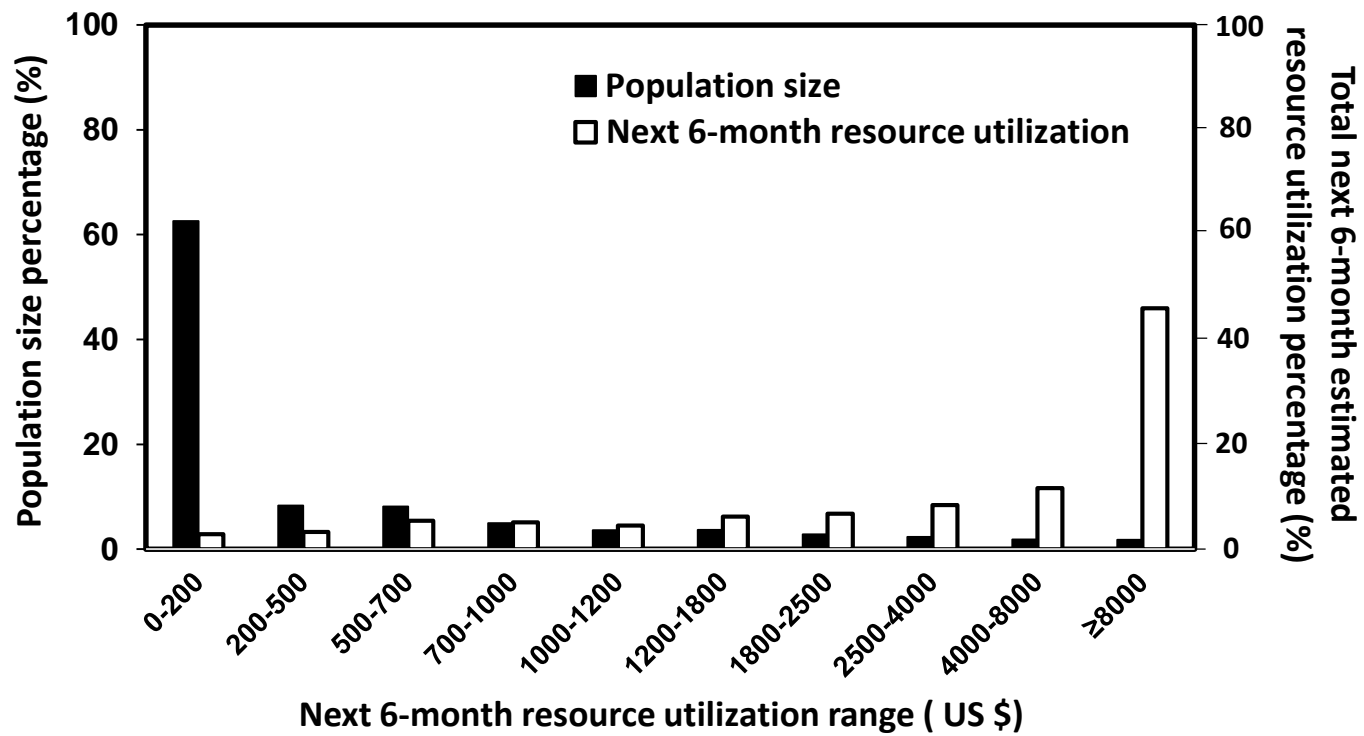

Supplement: Multimedia Appendix 3 [file jmir_v17i9e219_app3.pdf]
