# Supplementary material for: Online Prediction of Health Care Utilization in the Next Six Months Based on Electronic Health Record Information: A Cohort and Validation Study
Source: J Med Internet Res. 2015 Sep 22;17(9):e219. doi: 10.2196/jmir.4976 (PMC4642374; doi:10.2196/jmir.4976)
Supplement: Multimedia Appendix 4 [file jmir_v17i9e219_app4.pdf]

Multimedia Appendix 4. Case study of resource utilization patterns

The figure below shows resource utilization history on a female patient aged 59 years randomly selected from our study database. The monthly-based encounter history along with the health care resource utilization risk is displayed for this patient. For the period between October 2012 and September 2013, the patient had 5 inpatient admissions, 14 ED visits, and 36 outpatient visits. From the chart, the visit distributions were concentrated in the period from May 2013 to September 2013 (accounting for 80.0%, 4/5 of inpatient; 78.6%, 11/14 of ED; and 75.0%, 27/36 of outpatient visits), during which the risks underwent a dramatic increase from 36 to 84 (from the intermediate to high resource–utilization region). The correlation between the health care resource utilization risks and actual encounter volume demonstrates that our health care resource utilization risks could be incorporated with clinical, demographic, and utilization data to assist care providers in identifying patients in need of additional care interventions.

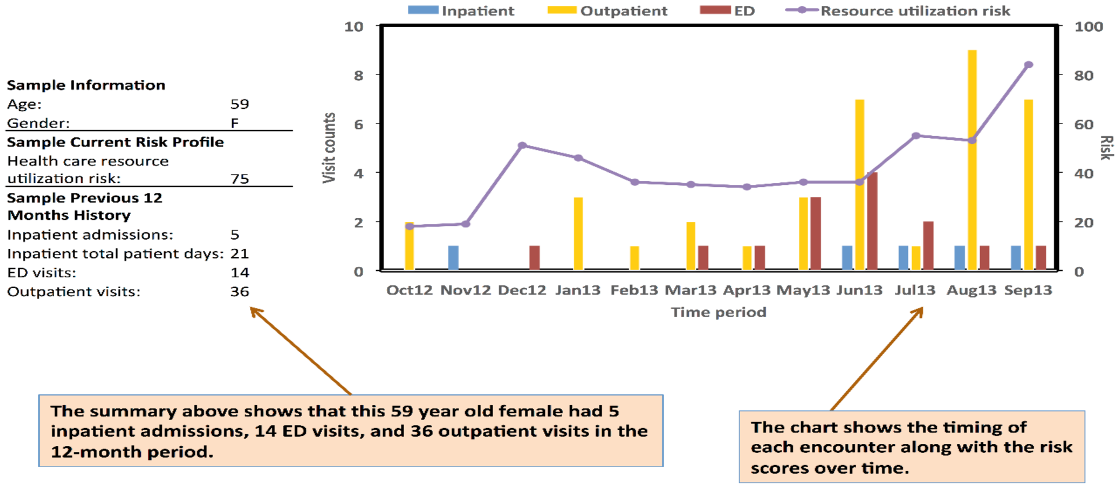

Figure. Twelve-month longitudinal study of a patient's visit counts and resource utilization risk values. Emergency department, inpatient, and outpatient visits and risks for health care resource utilization were summarized on a monthly basis from October 2012 to September 2013 for a patient randomly selected from our database.
