# Supplementary material for: Online Prediction of Health Care Utilization in the Next Six Months Based on Electronic Health Record Information: A Cohort and Validation Study
Source: J Med Internet Res. 2015 Sep 22;17(9):e219. doi: 10.2196/jmir.4976 (PMC4642374; doi:10.2196/jmir.4976)
Supplement: Multimedia Appendix 5 [file jmir_v17i9e219_app5.pdf]

Multimedia Appendix 5. System implementation of the online population-based risk model for health care resource utilization

A Spotlight Data Platform and analytic applications are live in production, to produce real time utilization risk on all patients throughout the Maine state. A fourteen machines virtual cluster was built to implement the Spotlight Data Platform integrating electronic medical records for analysis. Basically, all servers run as Window Server as operation system. Two failover SQL Servers perform as a central repository database for integrated data store and analytics data support, which make system stable and always on. An analytics engine built by HBI Solutions, Inc. works with the HIE database engine to produce utilization risk measure and would be refreshed on daily basis. A machine-learning server running with an open-source R engine for risk model development. Two scalable SharePoint servers are configured on IIS produce web-based population health and variation management applications to drive visualization dashboards of population risks.
