# Supplementary material for: Online Prediction of Health Care Utilization in the Next Six Months Based on Electronic Health Record Information: A Cohort and Validation Study
Source: J Med Internet Res. 2015 Sep 22;17(9):e219. doi: 10.2196/jmir.4976 (PMC4642374; doi:10.2196/jmir.4976)
Supplement: Multimedia Appendix 6 [file jmir_v17i9e219_app6.pdf]

Multimedia Appendix 6. Next 6-month clinical patterns of patients at different risk levels in the prospective cohort.

	Low (risk ≤ 30)	Intermediate (30 < risk ≤ 70)	High (risk ≥ 70)
Percentage of total population (%)	69.22	24.75	6.03
Percentage of total resource utilization (%)	33.25	34.64	32.11
Average cost per patient ($)	385.76	1,124.33	4,276.88
Percentage with age ≥ 65 (%)	18.54	25.58	41.35
Percentage with at least one inpatient admission (%)	1.35	9.25	28.55
Average number of inpatient admissions	0.02	0.12	0.47
Percentage with at least one emergency visit (%)	6.55	26.06	45.12
Average number of emergency visits	0.08	0.41	1.26
Percentage with at least one chronic disease (%)	8.67	51.32	83.92
Average number of chronic diseases	0.23	2.51	13.57
Percentage with hypertension (%)	2.62	11.25	30.35
Percentage with diabetes (%)	1.28	6.88	21.69
Percentage with heart disease (%)	1.89	6.96	25.91
Percentage with asthma or chronic obstructive pulmonary disease (%)	1.06	4.02	14.20
