# Supplementary material for: Online Prediction of Health Care Utilization in the Next Six Months Based on Electronic Health Record Information: A Cohort and Validation Study
Source: J Med Internet Res. 2015 Sep 22;17(9):e219. doi: 10.2196/jmir.4976 (PMC4642374; doi:10.2196/jmir.4976)

The dashboard showed the predicted risk of 6-month resource utilization for 1.4 million patients, and their corresponding characteristics.

The map showed where these patients originated.

The prediction was made on 07/21/2015

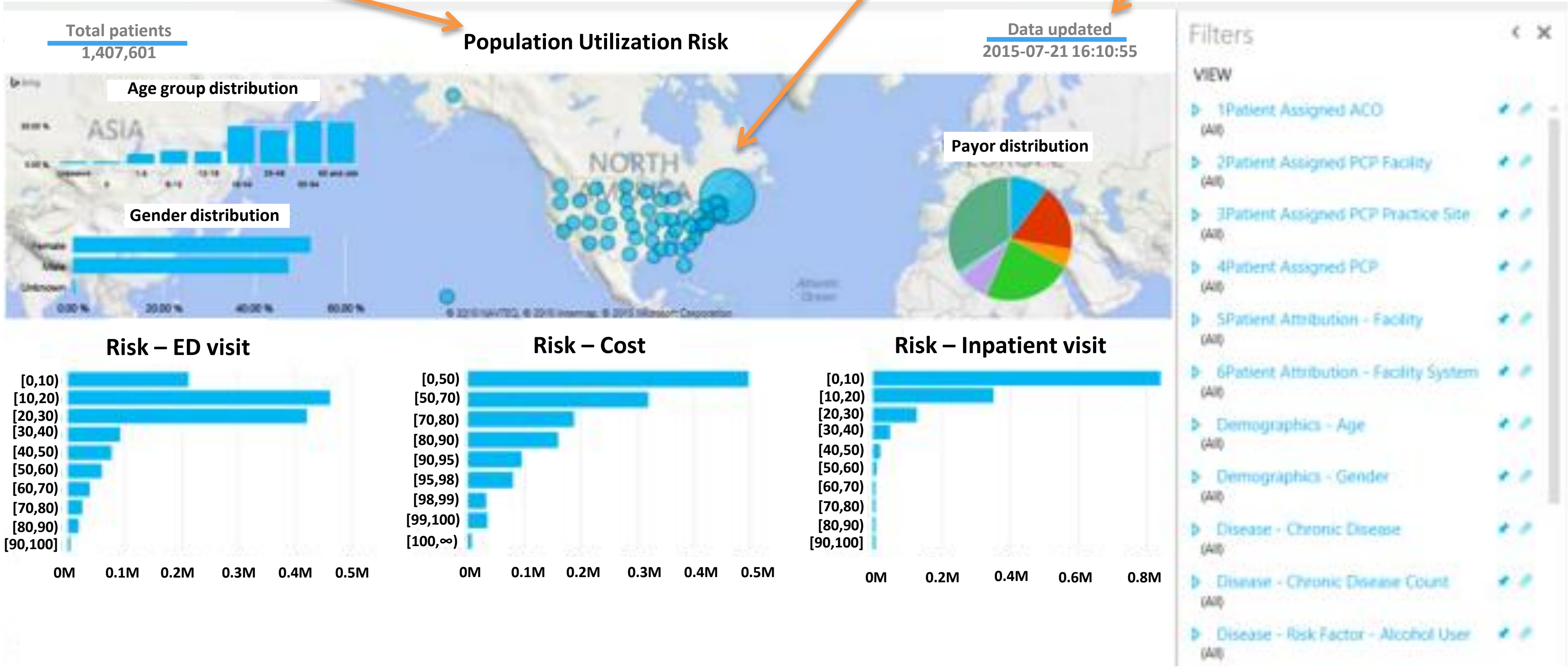

Supplement: Multimedia Appendix 7 [file jmir_v17i9e219_app7.pdf]
